# Supplementary material for: Resonant exciton transfer in mixed-dimensional heterostructures for overcoming dimensional restrictions in optical processes
Source: Nat Commun. 2023 Dec 9;14:8152. doi: 10.1038/s41467-023-43928-2 (PMC10710505; doi:10.1038/s41467-023-43928-2)
Supplement: Supplementary file 1 — Supplementary Information [file 41467_2023_43928_MOESM1_ESM.pdf]

Supplementary Information

Resonant exciton transfer in mixed-dimensional heterostructures for overcoming dimensional restrictions in optical processes

Nan Fang[1,*], Yih-Ren Chang[1], Daiki Yamashita[2,3], Shun Fujii[2,4], Mina Maruyama[5], Yanlin Gao[5], Chee Fai Fong[1], Keigo Otsuka[1,6], Kosuke Nagashio[7], Susumu Okada[5], Yuichiro K. Kato[1,2,*]

[1]Nanoscale Quantum Photonics Laboratory, RIKEN Cluster for Pioneering Research, Saitama 351-0198, Japan
[2]Quantum Optoelectronics Research Team, RIKEN Center for Advanced Photonics, Saitama 351-0198, Japan
[3]Platform Photonics Research Center, National Institute of Advanced Industrial Science and Technology (AIST), Ibaraki 305-8568, Japan
[4]Department of Physics, Keio University, Yokohama 223-8522, Japan
[5]Department of Physics, University of Tsukuba, Ibaraki 305-8571, Japan
[6]Department of Mechanical Engineering, The University of Tokyo, Tokyo 113-8656, Japan
[7]Department of Materials Engineering, The University of Tokyo, Tokyo 113-8656, Japan

* email: nan.fang@riken.jp, yuichiro.kato@riken.jp

Supplementary Note 1:
Morphology of suspended CNT/WSe_2 heterostructures

Using atomic force microscope (AFM), we have identified CNTs beneath the WSe_2 flake, as illustrated in Supplementary Fig. 1. 2D and 3D AFM images in Supplementary Fig. 1b,c are taken at the suspended region, which is marked in Supplementary Fig. 1a. Multiple CNT tubes and Fe catalyst can be seen above the substrate, which contributes to the rough surface of WSe_2 on the substrate. On the other hand, the suspended part of the WSe_2 flake exhibits uniformity characterized by minimal surface roughness, highlighting the cleanliness of our transfer process. Furthermore, this suspended section falls into the trench by approximately 6 nm, resulting in an intimate contact with the CNTs. As a result, two CNTs are discernible across the trench, as marked by arrows in Supplementary Fig. 1b. The height of the upper CNT is 1.05 nm, which is consistent with the CNT diameter discussed in this paper.

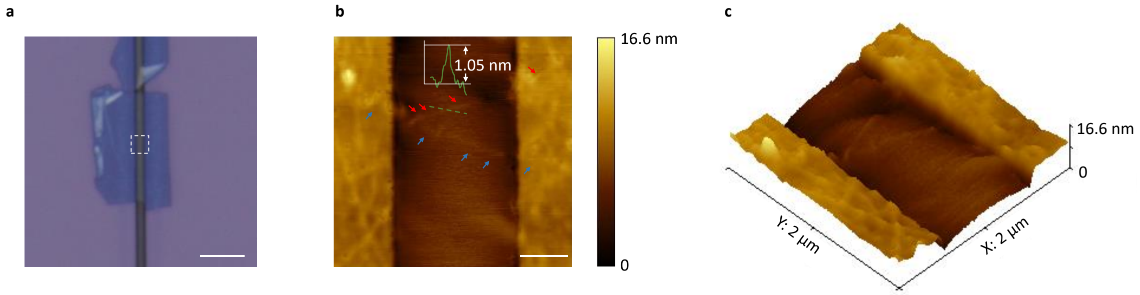

Supplementary Fig. 1 | Identifying CNTs in the heterostructure. a, An optical microscope image of a CNT/2L WSe_2 heterostructure. 2D (**b**) and 3D (**c**) AFM images for the area indicated by the white broken rectangle in (**a**). The inset in (**b**) is a line profile indicated by the green broken line. The scale bars are 5 and 0.4 µm for (**b**) and (**c**), respectively.

Supplementary Note 2:
Selective excitation through polarization

Because WSe_2 A excitons and CNT E_{22} excitons have different dimensionalities, the excitation process can be precisely controlled via the polarization angle. As shown in Supplementary Fig. 2a, the PLE map from a (14,3) CNT/3L WSe_2 heterostructure shows different behaviors at various excitation angles. Under the 80° excitation angle, which is parallel to the CNT axis, two excitation peaks corresponding to A and E_{22} excitons are observed. At the −10° excitation angle, the E_{22} excitation is entirely suppressed, leaving a single E_A peak in the PLE map. This profound selective excitation behavior can be explained by the excitation polarization dependence for A and E_{22} excitons, as indicated in Supplementary Fig. 2b. The E_{11} excitons show distinct 1D behavior as expected, irrespective of the excitation process, which is indicated by the emission polarization measurement in Supplementary Fig. 2c.

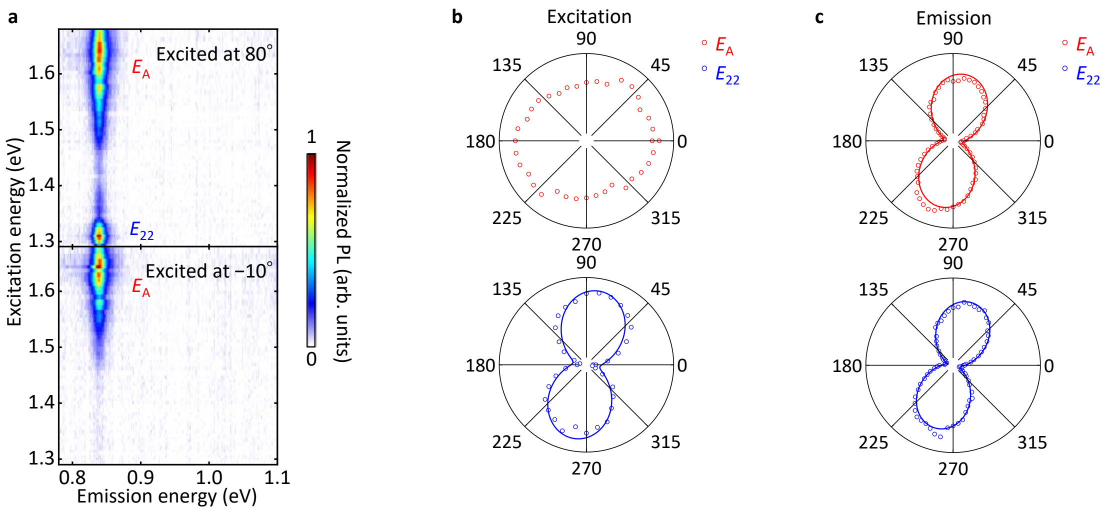

Supplementary Fig. 2 | Polarization-selective excitation process. a, PLE maps of the (14,3) CNT/3L WSe_2 heterostructure measured at 80° (upper) and −10° (lower) excitation angles, respectively. **b,** Excitation polarization dependence of PL from E_{11} states for E_A excitation (red circle) and E_{22} excitation (blue circle), respectively. **c,** Emission polarization dependence of PL from E_{11} states for E_A excitation (red circle) and E_{22} excitation (blue circle), respectively. The lines are fits to a cosine squared function.

Supplementary Note 3:
WSe_2 layer number dependence on exciton transfer process

The influence of the WSe_2 layer number on the exciton transfer process is examined in heterostructures comprising of (9,8) CNTs and WSe_2 flakes with varying thicknesses. As depicted in the Fig. 1c,d in the main text, the (9,8) CNT/1L WSe_2 heterostructure exhibits an efficient exciton transfer process. In Supplementary Fig. 3a, the E_A excitation peaks are also clearly observed in the heterostructures consisting of 3L and 4L WSe_2. In contrast, the E_A peak is considerably reduced in the sample with 10-nm-thick bulk WSe_2, presumably caused by the increased distance from the CNT to the surface of WSe_2 flake where excitons are generated.

The E_{11} and E_{22} energy shifts induced by different WSe_2 flakes are summarized in Supplementary Fig. 3b. It is surprising to observe that the 1L WSe_2 flake already substantially shifts both E_{11} and E_{22} energies through the dielectric effects [1]. Furthermore, 2L WSe_2 does not induce considerable shifts, indicating that the E_{11} and E_{22} excitons are already fully screened. The shifts in the thicker WSe_2 samples are mainly attributed to strain, which results in anticorrelated E_{11} and E_{22} energies shifts [2].

In Supplementary Fig. 3c, we plot the PL spectra from the suspended WSe_2 and the PLE spectra from the heterostructures. New low-energy PL peaks, attributed to the indirect excitons, emerge in thick WSe_2 flakes, with their intensity and peak energies significantly varying with the layer number [3]. In contrast, the energies of the A exciton emission peaks are relatively insensitive to the layer number. The PLE spectra also do not show distinct layer number dependence up to 4L, indicating that the exciton transfer process primarily occurs through the A excitons even in the samples with thick WSe_2 flakes.

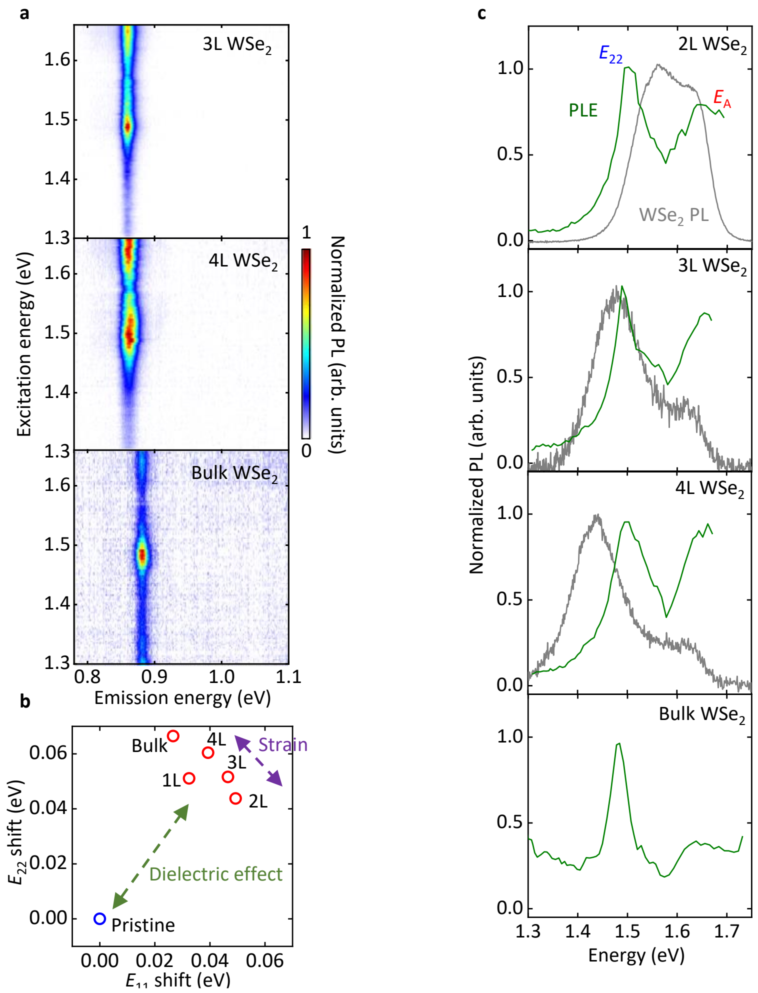

Supplementary Fig. 3 | Exciton transfer in heterostructures with varying WSe_2 thickness. a, PLE maps of the (9,8) CNT/3L, 4L and bulk WSe_2 heterostructures. The excitation power is 10 μW with the polarization aligned to the tube axis. **b,** E_{11} and E_{22} energy shifts in the samples with varying WSe_2 thickness. **c,** (green) Normalized PLE spectra of integrated E_{11} emission for the (9,8) CNT/2L, 3L, 4L, and bulk WSe_2 samples. (grey) PL spectra taken from the suspended WSe_2 flakes.

Supplementary Note 4:
A exciton lifetime in CNT/2L WSe_2 heterostructure

Compared to the 4L sample shown in Fig. 2a,b, 2L samples exhibit faster decay. Supplementary Fig. 4a,b illustrates the time-resolved measurements of E_{11} PL from a (12,4) CNT/2L WSe_2 heterostructure. Under E_A excitation, a slow decay curve is observed as in the case of 4L sample. We estimate the lifetime of the A exciton to be around 200 ps from fitting, which is shorter than the lifetime observed in the (9,8) CNT/4L WSe_2 heterostructure. Additionally, we notice the presence of another, slower decay component, which is absent in the data shown in Fig. 2b of the main text. This additional component could potentially be related to the dynamics of indirect excitons.

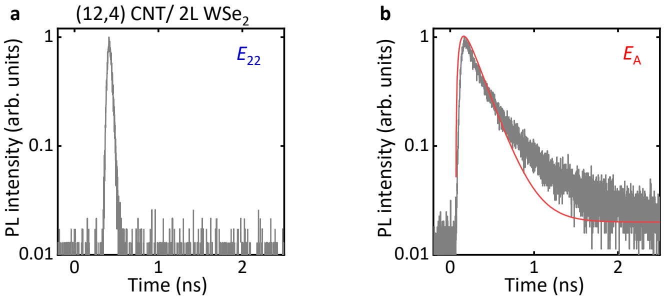

Supplementary Fig. 4 | The decay curve in a CNT/2L WSe_2 heterostructure. a, b PL decay curves taken from a (12,4) CNT/2L WSe_2 heterostructure for (**a**) E_{22} and (**b**) E_A excitation. Experimental results are indicated by gray lines. The red line is the fitting. The excitation power is 8 nW.

Supplementary Note 5:
A 1D-2D heterostructure with a long diffusion length

In some CNT/WSe_2 heterostructures with thick WSe_2 flakes, we find that the A excitons display long diffusion lengths. Supplementary Fig. 5a,b presents PL images for E_{22} and E_A excitation taken from an (8,6) CNT/3L WSe_2 sample. The noticeably enlarged PL image for E_A excitation indicates the long diffusion length. By fitting the line profile of the PL image as indicated in Supplementary Fig. 5c, we extract a diffusion length of 1.1 μm.

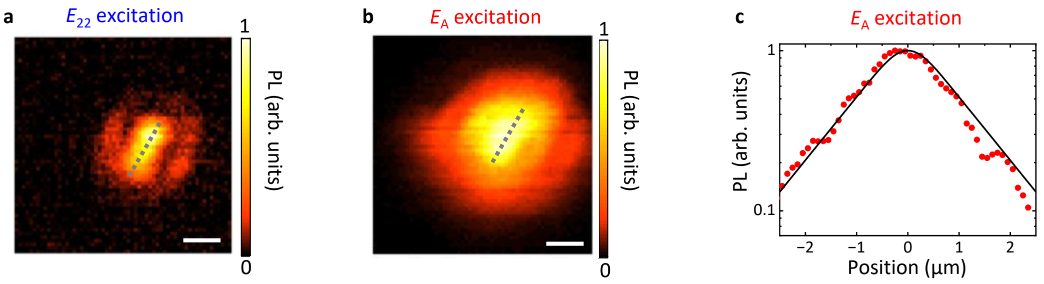

Supplementary Fig. 5 | PL images showing a long diffusion length. a,b PL intensity maps of E_{11} states for (**a**) E_{22} and (**b**) E_A excitation. The scale bar is 1 μm. The CNT is indicated by the broken gray lines. **c**, Line profile from the PL intensity map for E_A excitation. The excitation power is 10 μW. The CNT is indicated by the broken gray lines.

Supplementary Note 6:
EEA-limited efficiency factor *α*

E_{11} excitons in CNTs are known to undergo an efficient EEA process at high exciton densities, resulting in a sublinear power dependence of E_{11} emission. Given the high efficiency of the excitation through WSe_2 A excitons under resonant conditions, the EEA effect can be substantial. This will reduce the peak PL intensity and therefore α, making the apparent width of the E_A peak broader. Supplementary Fig. 6a and b depict the PLE spectra from the (10,5) CNT/1L WSe_2 heterostructure at various laser powers and the power dependence of the efficiency factor α, respectively. As the power decreases, the EEA effect is mitigated, and the E_A peak becomes more pronounced. The highest α value measured at low powers below 0.05 μW reaches 6.2, representing the intrisic exciton transfer process under resonant conditions.

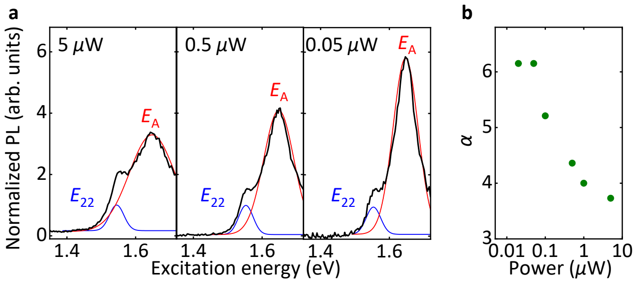

Supplementary Fig. 6 | Efficiency factor *α* at different laser powers. a, Normalized PLE spectra of integrated E_{11} emission for the (10,5) CNT/1L WSe_2 sample at different laser powers. The excitation polarization is aligned to the CNT axis. Black lines are experimental data, while red and blue lines are fits for E_A and E_{22} peaks, respectively. **b**, Laser power dependence of the efficiency factor α.

Supplementary Note 7:
Band alignment transition in 1D-2D heterostructures

Electronic band structure calculations on CNT and WSe_2 are performed within the framework of density functional theory implemented into the STATE package to examine the band alignment in the heterostructures. We use the generalized gradient approximation to describe the exchange-correlation potential among the interacting electrons. An ultrasoft pseudopotential generated by the Vanderbilt scheme is used to describe the interaction between electrons and ions. The valence wave functions and charge density are expanded in terms of the plane-wave basis set with cutoff energies of 25 and 225 Ry, respectively.

The calculated electronic band structures of CNTs and WSe_2 are plotted in Supplementary Fig. 7. The band structure of WSe_2 shows a clear dependence on layer number, with the valence band maximum at the Γ point being significantly more responsive than that at the K point. Within the heterostructure, it is important to note that the band offset of the valence band is considerably smaller than that of the conduction band. For a CNT with a small bandgap, such as the (13,0) CNT shown here, a type-I band alignment is formed in the heterostructure. With increasing the CNT bandgap through chirality alteration, the transition of the band alignment occurs for (8,0) CNT. Qualitatively, the transition from type-I to type-II with increasing bandgap is consistent with experimental results.

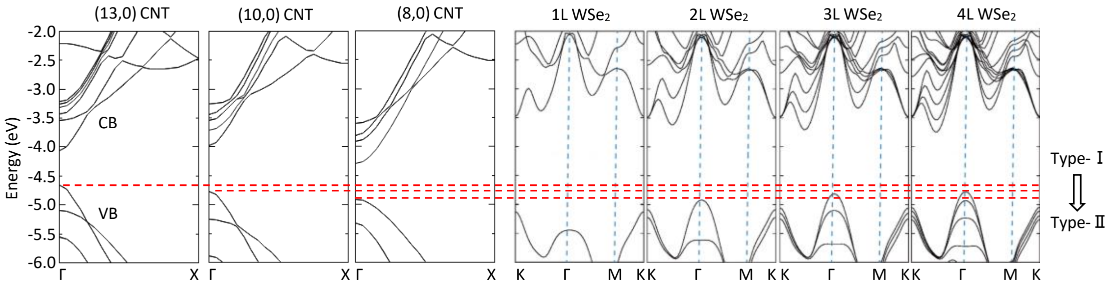

Supplementary Fig. 7 | Band alignment of CNT/WSe_2 heterostructures from DFT calculations. The calculated electronic band structures of (13,0), (10,0) and (8,0) CNT as well as 1L, 2L, 3L, and 4L WSe_2. The energies are measured from the vacuum level.

Supplementary Note 8:
Estimation of the transfer time

The efficiency factor α, which is defined by the ratio of E_A excitation efficiency with respect to E_{22} excitation, provides a way to estimate the exciton transfer time. The PL process is considered to be consisting of the following three steps. First, we define the dimension factor β to quantitatively capture differences in the proportion of light illuminating each material. For 2D materials, the light within the laser spot entirely illuminates the sample. In contrast, for (10,5) CNTs, given its diameter of 1.0 nm and a $1/e^2$ radius of the Gaussian laser spot r of 0.58 μm, only 1.4×10^{-3} of light interacts with the CNT. The factor β is calculated as $1/1.4\times10^{-3}$. Second, we determine the light absorption ratio, denoted as γ. The absorption for a monolayer WSe_2 flake at A exciton resonance is reported to be 10% [4]. For (10,5) CNTs, the absorption at E_{22} resonance is reported to be 44% [5]. γ is therefore estimated to be 10%/44%. Third, the excited A excitons need to transfer to the CNT, and we define T as the ratio of transferred excitons with respect to the total excited A excitons. Finally, α can be expressed as:

$$\alpha = \beta \times \gamma \times T.$$

For the (10,5) CNT/1L WSe_2 sample, where α is 6.2, this simple calculation leads to a T factor of 3.8×10^{-2}. If we igonre diffusion and assume that only the A excitons at the CNT position could transfer to the tube, the maximum value of the T factor should be $1/\beta = 1.4\times10^{-3}$ when all excitons are transferred. The transfer factor T here is over an order of magnitude larger, indicating that diffusion plays a major role in the exciton transfer process.

A Monte Carlo simulation is employed to gain more insight into the transfer process by including the exciton reservoir effect. The simulation considers exciton population profile in one dimension perpendicular to the CNT, taking into account WSe_2 A exciton generation, diffusion, and transfer processes over a short time interval Δt. During each interval, A excitons are generated using a Gaussian laser profile with a $1/e^2$ radius of 0.58 μm, where the peak center overlaps with the CNT. All existing A excitons are allowed to diffuse during the lifetime. The step size of a random walk is given by $\sqrt{2D\Delta t}$, where D represents the diffusion coefficient of A excitons, calculated using a lifetime of 90 ps [6] and a diffusion length of 600 nm. The exciton transfer takes place with a probability of $\Delta t/\tau_T$ when the exciton diffuses to the CNT/WSe_2 heterostructure region, with the boundary defined by the CNT diameter of 1.0 nm. Δt is set to be small enough to ensure $\sqrt{2D\Delta t} < 0.1$ nm for simulation accuracy.

Upon conducting the simulation, τ_T of 1.1 ps results in the factor T of 3.8×10^{-2}. Homo-dimensional heterostructures, such as TMD-based 2D-2D heterostructures and CNT-based 1D-1D heterostructures, exhibit a comparable transfer time of around 1 ps [7, 8]. In contrast, mixed-dimensional heterostructures, for example 0D-quantum-dots/2D-TMDs, usually have longer transfer times of 1−10 ns, arising from the increased distance between the materials due to complex interfaces [9, 10]. The fast decay observed here can

be attributed to the well-defined van der Waals interface in the mixed-dimensional heterostructure.

Sample	CNT chirality	CNT family	WSe_2 layer number	E_{11} (eV)	α	Laser power for α (μW)
#1	(8,6)	22	3	1.031	1.20	10
#2	(9,4)	22	1	1.102	0.00	10
#3	(9,4)	22	2	1.057	0.00	10
#4	(9,4)	22	2	1.115	0.00	5
#5	(9,4)	22	2	1.095	0.00	4
#6	(8,7)	23	2	0.970	0.00	5
#7	(10,5)	25	1	0.966	3.50	10
#8	(10,5)	25	2	0.973	2.98	10
#9	(10,5)	25	3	0.972	2.16	10
#10	(10,5)	25	4	0.957	3.10	10
#11	(11,3)	25	2	1.008	1.44	10
#12	(11,3)	25	3	1.006	1.43	10
#13	(12,1)	25	2	1.046	0.00	5
#14	(12,1)	25	3	1.014	0.00	5
#15	(9,7)	25	2	0.914	1.38	10
#16	(9,7)	25	3	0.908	1.19	10
#17	(9,8)	26	1	0.871	0.91	10
#18	(9,8)	26	2	0.855	1.24	10
#19	(9,8)	26	3	0.860	1.05	10
#20	(9,8)	26	4	0.862	1.38	10
#21	(10,8)	28	2	0.829	1.48	10
#22	(10,8)	28	3	0.838	0.46	10
#23	(10,8)	28	3	0.842	0.22	10
#24	(11,6)	28	2	0.867	0.74	10
#25	(11,6)	28	2	0.874	0.43	10
#26	(12,4)	28	2	0.923	1.13	10
#27	(12,4)	28	3	0.901	1.44	10
#28	(13,2)	28	3	0.929	0.00	10
#29	(14,0)	28	3	0.932	0.00	10
#30	(14,0)	28	4	0.946	0.00	10
#31	(12,5)	29	2	0.805	1.50	10
#32	(13,5)	31	2	0.841	0.17	10
#33	(14,3)	31	3	0.840	0.81	5
#34	(15,1)	31	3	0.862	0.53	10

Supplementary Table S1 | The efficiency factor α for all the samples.

Supplementary References

S1. Fang, N., Otsuka, K., Ishii, A., Taniguchi, T., Watanabe, K., Nagashio, K. & Kato, Y. K. Hexagonal boron nitride as an ideal substrate for carbon nanotube photonics. *ACS Photonics* **7**, 1773 (2020).

S2. Huang, M., Wu, Y., Chandra, B., Yan, H., Shan, Y., Heinz, T. F. & Hone, J. Direct measurement of strain-induced changes in the band structure of carbon nanotubes. *Phys. Rev. Lett.* **100**, 136803 (2008).

S3. Zhao, W., Ghorannevis, Z., Chu, L., Toh, M., Kloc, C., Tan, P.H. & Eda, G. Evolution of electronic structure in atomically thin sheets of WS_2 and WSe_2. *ACS Nano* **7**, 791 (2013).

S4. Kim, H., Ahn, G. H., Cho, J., Amani, M., Mastandrea, J. P., Groschner, C. K., Lien, D. H., Zhao, Y., Ager III, J. W., Scott, M. C., Chrzan, D. C. & Javey, A. Synthetic WSe_2 monolayers with high photoluminescence quantum yield. *Sci. Adv.* **5**, 4728 (2019).

S5. Malapanis, A., Perebeinos, V., Sinha, D. P., Comfort, E. & Lee, J. U. Quantum efficiency and capture cross section of first and second excitonic transitions of single-walled carbon nanotubes measured through photoconductivity. *Nano Lett.* **13**, 3531 (2013).

S6. Cadiz, F., Robert, C., Courtade, E., Manca, M., Martinelli, L., Taniguchi, T., Watanabe, K., Amand, T., Rowe, A.C.H., Paget, D. & Urbaszek, B. Exciton diffusion in WSe_2 monolayers embedded in a van der Waals heterostructure. *Appl. Phys. Lett.* **112**, 152106, (2018).

S7. Kozawa, D., Carvalho, A., Verzhbitskiy, I., Giustiniano, F., Miyauchi, Y., Mouri, S., Castro Neto, A. H., Matsuda, K. & Eda, G. Evidence for fast interlayer energy transfer in $MoSe_2/WS_2$ heterostructures. *Nano Lett.* **16**, 4087 (2016).

S8. Koyama, T., Asaka, K., Hikosaka, N., Kishida, H., Saito, Y. & Nakamura, A. Ultrafast exciton energy transfer in bundles of single-walled carbon nanotubes. *J. Phys. Chem.* **2,** 127 (2011).

S9. Prins, F., Goodman, A. J. & Tisdale, W. A. Reduced dielectric screening and enhanced energy transfer in single-and few-layer MoS_2. *Nano Lett.* **14**, 6087 (2014).

S10. Tanoh, A. O. A., Gauriot, N., Delport, G., Xiao, J., Pandya, R., Sung, J., Allardice, J., Li, Z., Williams, C. A., Baldwin, A., Stranks, S. D. & Rao, A. Directed energy transfer from monolayer WS_2 to near-infrared emitting PbS–CdS quantum dots. *ACS Nano* **14**, 15374 (2020).
